# The Quality of Life of Iranian Mothers and Fathers of Children with Autism

**DOI:** 10.3390/children12040454

**Published:** 2025-03-31

**Authors:** Sayyed Ali Samadi, Farzaneh Ghanimi, Roy McConkey

**Affiliations:** 1 Institute of Nursing and Health Research, University of Ulster, Belfast BT15 1AP, UK; s.samadi@ulster.ac.uk; 2 Deputy of the Daily Rehabilitation Centre Section, Iranian State Welfare Organization (ISWO), Tehran 25529, Iran

**Keywords:** quality of life, mothers, fathers, family, autism, Iran, WHOQOL-BREF, stress, coping, caregiving

## Abstract

Autism is a complex neurodevelopmental condition that has life-long impacts on individuals and their families, who are the main caregivers. This study investigated the self-reported quality of life (QoL) of Iranian mothers and fathers and aimed to identify the predictors associated with higher scores on the World Health Organisation’s QoL brief measure (WHOQOL-BREF), particularly those related to their child’s autism. Methodology: A cross-sectional study was conducted with two national samples of parents: one in which the mothers and father were related (n = 119 families) and a second sample involving 383 unrelated mothers and fathers, making a total of 623 Iranian parents of children diagnosed with autism spectrum disorder (ASD). Participants completed self-report measures assessing QoL, perceived child-rearing stress, social support, and coping strategies as well as pertinent demographic information of themselves and their child. These were chosen on the basis of previous research. Data were analysed using descriptive statistics and linear regression analysis. Results: There were few significant differences between mothers and fathers in both samples on the various rating scales. Regression analyses identified satisfaction with personal health and with their marriage, along with support from family as the main predictors of higher quality-of-life ratings. Access to professionals and positive interactions with their children augmented the contribution to parents’ overall quality of life. The children’s autism had minimal direct impact on the parental quality of life although it likely accentuated the importance to parents of the identified predictors. Discussion: The findings support the case for services to adopt a family-centred approach to the support they provide and for a greater engagement with fathers. Three strands are proposed for future research: recruiting families who receive little or no support, the identification of strategies for meeting the personal needs of parents, and undertaking longitudinal studies of interventions to improve family quality of life and the outcomes these have on the person with autism.

## 1. Introduction

Autism spectrum disorder (ASD) is a complex neurodevelopmental condition that significantly impacts individuals and their families and caregivers [1,2,3]. Their social and emotional difficulties have been well-documented internationally which make parental caregiving especially challenging, with mothers reporting poorer physical and mental health. Higher levels of stress, anxiety, and depression have been reported especially among mothers [4] along with physical fatigue and burnout [5]. Financial strain and the costs associated with therapies, specialized education, and medical care can be significant [6]. Finally, social isolation due to caregiving responsibilities can limit social interactions and lead to feelings of isolation [7].

Previously, the assumption was parental stress and ill-health emanated from their child’s behavioural and emotional difficulties. However, longitudinal research suggests that the relationship is bi-directional, in that parental health makes their children more prone to exhibit behavioural and emotional problems [8]. Up to now, research has focused more on understanding the characteristics of autism from early childhood through to adulthood, while the psychological and emotional impact on caregivers often remains understudied [9,10]. This oversight is particularly concerning in countries like Iran, where cultural and societal factors can exacerbate the challenges faced by families [11,12,13].

Research into the experiences of mothers and fathers of children with autism is essential for several reasons. Firstly, it sheds light on the unique stressors and challenges that these parents may encounter, such as increased levels of stress, anxiety, and depression [14,15]. Secondly, it helps to identify protective factors, such as social support and effective coping strategies, that can mitigate these negative impacts [16]. Thirdly, it informs the development of targeted interventions and support services that can improve the well-being of both caregivers and individuals with autism [17,18,19]. For example, a child’s behavioural issues may be compounded by parental, mental ill-health [20]. Hence, interventions need to address parental issues and not focus solely on the child.

Family quality of life is an overarching concept that usefully combines the needs of the individual with autism and those of the wider family members. A systematic review of 12 studies, with mostly mothers of children with autism aged up to 18 years, reported poorer QoL among these parents compared to parents of typically developing children or to population norms [21]. The variables that were commonly associated with lower parental QoL included child behavioural difficulties, unemployment, being a mother, and lack of social support.

A more recent review of 15 studies undertaken in 10 different countries [22] confirmed the lower quality of life of parents with autism particularly in their physical, psychological, and social health and in spirituality, and the deleterious impact of a child’s behavioural issues. By contrast, better quality of life was associated with less severe autism as was increased family income, a higher level of parent education, and access to social supports. Again, the informants were mostly mothers. However, a study involving 130 matched pairs of mothers and fathers found few differences between fathers and mothers on the impact on the child’s autism on their quality of life [23].

A transnational study involving seven countries examined the quality of life of 1121 parents of children with autism [24]. The countries varied in the way the various domains of quality of life were rated, which, the authors suggested, illustrated cultural differences in how personal wellbeing was construed. They called for greater attention to be paid to socio-cultural influences in future research and more reports from low- and middle-income countries.

To date, research into family quality of life has been concentrated on high-income, English-speaking countries. Yet, the design of effective, family-centred interventions needs to be attuned to the family culture and the delivery of care to members with additional needs. The nature of caregiving can vary significantly across cultures due to differences in family structures, social support systems, healthcare systems, and cultural beliefs about aging and disability [25]. By examining different cultural contexts, researchers can identify specific challenges faced by caregivers in different societies, such as language barriers, cultural misunderstandings, and limited access to resources [25,26]. Descriptive studies across different cultures would assist in developing culturally sensitive interventions and tailored support to families and to their dependents [27]. By recognizing cultural nuances and communication styles, healthcare providers and social workers can improve communication with caregivers from diverse backgrounds, leading to better care outcomes [28].

### Aims of the Study

This present study aims to address the gaps in current knowledge around family quality of life. First, the focus is on the quality of life of Iranian families caring for a son or daughter with autism from childhood into adulthood. Unlike more developed nations, there is little prospect of persons with autism finding alternative full-time support outside of the family. Second, in other cultures, mothers and women in general play a subservient role to men. Yet, the role of fathers and the impact on them from caregiving has been a secondary consideration in a lot of past research internationally, with conflicting evidence about the extent to which their experiences are different or similar to that reported by mothers [29]. Third, the diverse influences on family quality of life identified in previous research need to be considered together in order to identify those that have the greater influence on the family life. This necessitates a larger sample of participants from across the country than has been previously recruited in small-scale, localised studies.

Thus, the main aim of this descriptive study was to identify the major determinants on Iranian mothers’ and fathers’ reports of their quality of life. Those studied included child, parental, and family characteristics alongside parental health, stress, coping strategies, and access to formal and informal supports; all of which have been implicated in previous research.

Uniquely, the study recruited two samples of parents from all 31 provinces in Iran. One sample of 119 families obtained reports from both mothers and fathers, which implicitly controls for child and family characteristics that may affect parental ratings of QoL. A second sample obtained reports from 224 mothers and 161 unrelated fathers drawn from the same centres. In all, 623 parents participated in the study.

## 2. Method

In the absence of any formal ethical procedures in Iran for research outside of universities, ethical considerations based on the WMA Declaration of Helsinki on Medical Research involving Human Subjects revised on 19 October 2013 [30] were fully observed, and the arrangements were formally approved by the Iranian Social Welfare Organization (ISWO). Caregivers were provided with a written comprehensive information sheet and a consent form that detailed the purpose of the study, the specific procedures involved, and the potential risks and benefits, as well as their right to withdraw from the study at any point without any impact on the services they were receiving. Their written informed consent was obtained by their signature on the designated section of the questionnaires.

### 2.1. Context

The parents were recruited through the daycare centres for children and young adults with autism under the supervision of the Iranian Social Welfare Organization (ISWO). These centres provide educational and rehabilitation services using multi-disciplinary teams of psychologists, therapists, social work, and educationalists. They are privately managed, and fees are charged according to family means.

Out of 70 active, autism daycare centres across the country, 63 (90%) were considered, at least one from each of the 31 provinces. To encourage parent participation, an information sheet was distributed to suitable families in coordination with the centre managers. The inclusion criteria for parents in this study were the following: caregiving for a child with autism at the time of the study, minimum education level of 9th grade, and willingness to participate in the research. The exclusion criteria included chronic physical illnesses such as cardiovascular diseases, kidney diseases, diabetes, AIDS, as well as the use of psychotropic medications, drug addiction, or other traumatic events during the research period and the previous year. It was indicated that both parents should separately take part.

### 2.2. Study Sample

At the time of the study, there were 1533 individuals registered in the selected autism centres, and an estimated 90% were sent invitations to join the study. In all, 660 parents agreed to participate, but 37 cases were excluded due to incomplete completion of the questionnaire or family circumstances, leaving a final sample size of 623 self-selected parents (an estimated 23% response rate). Since their sons or daughters were from autism centres under the supervision of the ISWO, they were all officially diagnosed as having autism by the ICF Commission of ISWO in their provinces.

Table 1 presents the demographic details of the parents within the two samples as reported by respondents in a self-completion questionnaire devised for the study that drew on likely predictors of quality of life. Statistical tests were used to determine the significance of differences between mothers and fathers in each sample as shown in the Table 1. In both samples, fathers were older than mothers and much more likely to be employed, but in the related sample only, fathers were more satisfied with their health. Mothers in the unrelated sample showed more dissatisfaction with their marriage. On all other indicators, there were no differences between mothers and fathers.

Table 2 presents details of the persons with autism in their family as reported by respondents on the demographic questionnaire they completed.

Across all parents, the mean age of their child was 12 years but variously ranged from 4 to 44 years. Around three-quarters were male with around half capable of verbal communication, although this was higher for those in the sample of related parents. Parents reported upwards of two-thirds of their children had behavioural issues, and around 40% had sensory issues with no significant differences between ratings of fathers and mothers. Around half the parents rated these issues as moderate, and although there were no differences between mothers and fathers, fewer children in the related parents sample rated their children’s difficulties as severe.

### 2.3. Questionnaires Used

A number of well-established, short-form questionnaires were identified and which had featured extensively in domestic and international research.

In order to establish the construct validity and internal reliability of the questionnaires when used with the chosen samples, factor analyses were undertaken of each using the total number of respondents in both studies (n = 623). Further details are available on request from the authors.

#### 2.3.1. World Health Organization Quality of Life Scale “WHOQOL-BREF” [31]

This questionnaire has been used extensively in many different countries including Iran [32], where a study with nearly 6000 cases had confirmed its psychometric properties. Quality of life is defined by WHO “*as individuals’ perceptions of their position in life in the context of the culture and value systems in which they live and in relation to their goals, expectations, standards and concerns*”.

The questions were taken from a more extensive tool that was designed to represent different domains that contribute to people’s quality of life, namely physical health, psychological, social relationships, and environment. Respondents are asked to rate how much they have experienced certain things in the last two weeks. A five-point Likert scale is presented for each item and responses start with “Not at all: A little: A moderate amount; Very much: Extreme Amount”. Scores are assigned from 1 to 5 with higher scores reflective of a higher quality of life (with certain items being reverse coded because of their wording).

The factor analyses on the parental data failed to replicate the four distinct domains underpinning the questionnaire. Rather, 21 of the 26 items loaded on one main factor which accounted for 40% of the variance along with three further factors which together added 18% additional variance. On all factors, the items were drawn from two or more of the domains. Moreover, most items loaded on two or more of the factors. Hence, it was decided to compute one total score calculated across 23 items. Scores could range from 23 to 115 with higher scores indicative of a better quality of life.

Cronbach’s Alpha was used to assess the internal reliability, and for this indicator of quality of life, it was 0.933.

Two further items from the questionnaire were also used that employed different rating scales. One was Item 2: “How satisfied are you with your health”, with ratings starting with “very dissatisfied to very satisfied” and scores ranging from 1 to 5. The other was Item 26 which refers to feelings in the last two weeks: “How often do you have negative feelings such as blue mood, despair, anxiety, depression”. Ratings ranged from “Never to Always”. Scores ranged from 1 to 5 with higher scores indicative of poorer emotional health (see Table 1).

#### 2.3.2. Perceived Stress Scale (PSS) [33]

This widely used 18-item scale is intended to assess stress associated with child-rearing as it had previously been translated into Farsi [34] with a very large sample of parents of children with intellectual and developmental disabilities [35]. It consisted of two domains: positive and negative aspects of parenting. Sample items include “enjoy spending time with my child(ren)” (positive) and “It is difficult to balance different responsibilities because of my child(ren)” (negative). Respondents used a 5-point Likert scale where 1 represents strongly disagree, 2 is disagree, 3 is undecided, 4 is agree, and 5 is strongly agree.

A factor analysis with the Iranian sample confirmed two main factors with factor 1: positive (34% variance) and factor 2 negative (14%). Only 14 of the 18 items loaded on these two factors which were then used in computing total scores for parental ratings in this study. The Cronbach Alphas were 0.798 for positive parenting and 0.818 for negative parenting experiences.

#### 2.3.3. Multidimensional Scale of Perceived Social Support “MSPSS” [36]

This 12-item scale has been used to assess the support provided to persons from three sources: (a) family (e.g., “I can talk about my problems with my family”); (b) friends (e.g., “I have friends with whom I can share my joys and sorrows”); and (c) significant other (e.g., “There is a special person in my life who cares about my feelings”). Each item was rated on a 5-point, Likert-type scale ranging from strongly disagree (1) to strongly agree (5). The scale had been used previously in Iran using the three factors [37]. However, with the present sample, a two-factor solution emerged: family plus special person (54% variance) and friends (14%), the Cronbach Alpha = 0.913 for factor 1 and Alpha =0.891 for factor 2. Total scores were calculated across eight items for family and special person (range 8 to 40) and four items for friends (range 4 to 20). The correlation between the two factor scores was r = 0.572.

A further measure was added in terms of professional supports that were available to the family, namely a count of the number of different professionals that were accessed in the last year by the family and child, such as consultations with psychiatrist, centre staff, occupational therapist, speech therapist, other therapies (for instance, music therapy), and others (as named by respondents). For this sample, the scores ranged from 1 to 6.

#### 2.3.4. Multidimensional Coping Inventory (MCI) [38]

Numerous studies have been conducted internationally using this scale which consists of 48 items and assesses three coping styles which were originally designated as “task-oriented”, “emotion-oriented”, and “avoidance-oriented” coping. Example items are the following: “think about the event and learn from my mistakes” (Task); “feel anxious about not being able to cope” (emotion); and “go to a party” (avoidance). Items are rated on a five-point scale from “never to always”. The scale had been previously used in Iran [37]. However, a factor analysis of the ratings given by parents in this study did not confirm the three types of coping as indicated. Each factor contained a mix of items from three different coping styles. However, the items did have a face validity, so Cronbach Alphas were calculated for the items that are thought to be indicative of different coping styles. They were the following: task = 0.658; avoidance = 0.642; emotional = 0.749, which are similar or better to that previously reported for an Iranian sample 0.64, 0.61, and 0.60, respectively [37]. Although the alphas were somewhat weak, total scores were nonetheless calculated for each respondent’s use of each coping strategy. Across the 16 items for each strategy, these could range from 16 to 80.

### 2.4. Procedure

Parents who volunteered to take part in the study were invited to a meeting at the centre attended by their son or daughter. The printed questionnaires were explained to families by a centre staff member. In some cases, responses were provided in person at the centre, but mostly parents took the questionnaires home and personally returned them to the centre after completion. A staff member reviewed with parents any issues or concerns that had arisen when completing the questionnaires and assurances of confidentiality were repeated. Few concerns were raised, and the parents seem to have valued the opportunity for them to express their views and experiences.

The information was recorded by the first two authors in an Excel file that was later transferred to the Statistical Package for Social Sciences (SPSS) version 27 for data cleaning and analysis. The analyses were conducted separately for the two samples of parents using parametric and Chi Square Tests, or Analysis of Variances to identify relationships between the various predictors and the quality-of-life measure. Those which had a statistically significant relationship (*p* < 0.05) were then entered into a linear regression to identify those that contributed to the regression model while controlling for the inter-relationships among the predictor variables.

## 3. Results

Table 3 summarises the mean scores on the questionnaire measures described above for the two samples. In addition, Chi Square tests were used to assess the statistical significance differences between mothers and fathers. The outcomes are noted in the table.

In both samples, fathers rated their quality of life higher than did mothers. However, there were no differences in their ratings of supports from family, friends, or professionals. Likewise, both sets of parents rated themselves similarly on positive and negative stress in child-rearing. Unrelated fathers had higher scores on emotional and task coping strategies but not on avoidance coping. There were no significant differences between related mothers and fathers in the use of coping strategies.

Table 4a presents the correlations between the predictor measures and quality-of-life ratings for the two samples. There were small but statistically significant relationships between the three forms of support and with positive and negative coping (in the latter the lower scores are linked with higher quality-of-life scores). However, the coping scores were not associated with quality-of-life scores except in the case of avoidance coping for related parents.

A similar analysis was undertaken to determine if the child and family characteristics were associated with quality-of-life scores. One-way analyses of variance were used to determine the significance of differences. Table 4 summarises those that were significant, notably parental satisfaction with their health and with their marriage and education level and employment.

However, other characteristics were not significantly associated with quality-of-life scores: parental age, income, type of marriage, negative mood, or number of children. Also, the age of the child with autism, their gender, and verbal communication did not affect the quality-of-life ratings.

### Regression Analyses

The above predictors that had a significant relationship with parental quality-of-life scores were included in a linear regression analysis to identify those that contributed to the regression model while controlling for the inter-relationships among the predictor variables. This was done in two stages in order to reduce the number of variables in the regression given the size of the samples. Separate regression analyses were first undertaken on the various measures identified in Table 4a and for the parent and child characteristics listed in Table 4b. The variables that had contributed significantly (*p* < 0.05) to either of these regression models were then included in a further regression analysis. Table 5a summarises the final analysis for related parents.

The resulting regression model for the quality of life of related parents was significant (Analysis of Variance: F = 16.71, *p* < 0.001) and accounted for around 40% of the variance. Higher quality of life was associated with parents’ satisfaction with their marriage and their health and having support from family and a special person. There was a tendency (*p* < 0.10) also for those who had access to more professionals and who scored higher on positive child care experiences to have higher quality-of-life scores. Nonetheless, a sizeable proportion of the variance on quality-of-life scores remained unexplained.

The regression model of unrelated parents was also significant (F = 20.97, *p* < 0.001) but accounted for a lower amount of the variance around 34%. For this sample too, a higher quality of life was associated with parental satisfaction with their marriage and their health, as was having attended higher education, having access to professionals, along with more positive child-rearing experiences. There was tendency (*p* < 0.10) for higher quality-of-life scores to be related to having children with less behaviour issues and having support from friends. As noted above, a larger amount of variance on quality-of-life scores remained unexplained within this sample, possibly due to the greater heterogeneity of this sample of unrelated parents.

Finally, it is worth noting that for both samples, the gender of parents, their educational level, employment, income, family size, and negative mood were not related to their quality-of-life ratings.

## 4. Discussion

This study had two notable strengths. First, two large samples of parents were recruited from across Iran which uniquely involved a matched sample of mothers and fathers from the same family as well as additional mothers and fathers from different families. This enabled the findings to be replicated in two different samples. The study uniquely addressed a significant gap in research by including a sizeable number of fathers, enabling meaningful comparisons between mothers and fathers. Second, the large samples enabled a variety of influences that previous studies have identified to be investigated on parental ratings to their quality of life while controlling for the inter-relationships among them which previous research has been unable to do.

The findings in relation to the gaps in the current literature are worth highlighting. Firstly, the three main predictors of Iranian parents’ ratings of their quality of life were the satisfaction with their health and with their marriage and having support from their families. Other influences were less effective but did augment the contribution to parents’ overall quality of life, such as access to professionals and positive experiences with their children. By contrast, behavioural issues or other autistic features of the children had little influence on these parents’ quality of life in contrast to past research with families caring for children with autism [21].

Secondly, the differences between fathers and mothers were limited to a few characteristics such as the older age of fathers and being employed. Although fathers in related families rated themselves as having better health, and those in unrelated families had higher satisfaction in their marriage, these did not feature as significant influences on their quality-of-life ratings, in that gender did not contribute significantly to the regression models. Perhaps this is a reflection of Iranian culture that fathers are expected to be more supportive of the family and its weaker members in particular [39].

Thirdly, although poorer health was a dominant predictor of quality of life in the Iranian general population [40], other variables that had been found to be associated with quality of life in Iran as well as internationally did not impact the parent’s ratings in this study, such as educational level, employment, income, and family size. This finding apparently goes against the extant literature on the social determinants of health that has identified social and economic circumstances as having a significant influence on parental wellbeing [41]. Rather, it seems when Iranian parents are raising a child with autism this in itself becomes a more dominant determinant of parental health that then reduces the impact of other socio-economic influences. Indeed, the poorer health experienced by parents of children with autism is well attested in the literature [42]. Furthermore, our findings suggest that the presence of a child with autism prompts parents to identify other determinants of their quality of life such as enjoying a satisfactory marriage, receiving support from families, and having access to professionals. But when these too are diminished or absent, then the quality of their lives is diminished despite the parents’ social and economic status which are predictors of quality of life in population studies. Latterly calls have been made for greater attention to be paid to the social dimension of the determinants of health and quality of life [43] which these findings would endorse.

### 4.1. Implications for Support Services

Our findings have implications for the supports provided by clinicians and centres to Iranian families in particular but also regionally if not internationally. They augment the case for primary care and specialist services to adopt a family focus and not solely a child focus to their supports [44]. In particular, support services should encourage fathers as well as mothers to engage in the interventions to assist the child as well as supporting each other. Likewise, they should encourage the parents to seek support from other family members such as grandparents and siblings, but if they are not available or willing to assist, then from friends. Centre staff could encourage friendship networks among parents attending the same facility and living in the same neighbourhood. Such parent-to-parent initiatives have proved to be very helpful to many but admittedly not to all parents [45].

A further dimension that primary care and specialist services should consider is attending to the health needs of mothers and fathers. At a minimum, this might consist of enquiring about their physical and emotional wellbeing and attentively listening to their responses. A further step could be giving them information about available services in their locality or providing some written or internet resources for them. More direct support could come from creating opportunities from groups of parents to meet over a number of sessions to focus on issues such as stress and coping strategies. Often fathers miss out on such sessions, but having “men-only” sessions in evening time with male facilitators, if available, has been a fruitful strategy [46].

Another boost to family quality of life comes through promoting more positive parent–child interactions by identifying mutually enjoyable activities for use at home and advice on minimizing any behavioural or sensory issues or sleep problems [47]. Visits to the family home will help professionals to attune their advice and plans to the physical and social environment of the home.

The parents in this study do not seem to use a consistent coping strategy, particularly the use of a task—or problem-solving approaches—which have been more productive rather than emotional or avoidance strategies [23]. Indeed, only 13% of parents in this study used the former to a greater extent than the other two strategies. Exploring with parents individually, or in small groups, the strategies they currently use and promoting the use of task-focused strategies is a further development to the current practices by professional staff in centres and clinics.

### 4.2. Further Research

The limitations of this study could be addressed in future research. The samples obtained may be biased towards families who are already experiencing a reasonable, if not a good quality of life, whereas those who face more challenges in their lives may have been reluctant to volunteer to take part in this study. Additionally, there are many more families who have no contact with centres or access to professional guidance and advice which is especially common in low-income countries. They too may have a much poorer quality of life although environmental factors such as poverty, poor housing, and inadequate nutrition are likely to have a greater impact [48]. Nevertheless, it is especially challenging to recruit such parents, but not impossible, for example using snow-ball sampling which is when parents identify other families known to them. Likewise public health and primary care staff may be aware of families whom they could approached; ideally through personal invitations by phone or personal contact. In addition, replicating this study in other cultures would be advantageous [24].

Alternatives measures of quality of life are also available, especially those tailored to the needs of parents who have a child with a disability [49]. Likewise, there are other scales relating to parental stress, coping, emotional wellbeing, and family functioning that could be used or adapted for use in clinical practice, especially those that have been translated into Farsi or other languages [50,51]. A further study could assess promising scales for use by practitioners in terms of their face validity and brevity to reduce the demands placed on respondents.

Further research could be useful to explore the relationships between improvements in family quality of life and outcomes for the children or adult with autism. In particular, a longitudinal study that monitors the impact of a family-based intervention over a two to three-year period would provide valuable insights [20].

In conclusion, conceptual frameworks such as family quality of life and social determinants of health can be used by professional services in primary care and health and social care agencies to extend their support to parents and in turn to the child or adult with autism or other life-long cognitive impairments. Nonetheless, their staff will require additional training and changes to their current roles and practices for this to happen.

## Figures and Tables

**Table 1:** Parental demographic information, frequencies, and percentages by gender and the statistical comparisons between mothers and fathers in the unrelated and related samples. NS = Not Significant.

Variable	Unrelated Parents		Related Parents	
	Mothers (N = 224)	Fathers (N = 161)	Mothers (N = 119)	Fathers (N = 119)
Parents’ age in years:	Mean: 40.8 SD.: 7.12 Min: 24.00 Max: 72.00	Mean: 45.0 SD.: 8.25 Min: 29.00 Max: 75.00	Mean: 38.8 SD.: 6.95 Min.: 26 Max: 63	Mean: 43.7 SD.: 6.62 Min.: 31 Max: 61
	*F = 29.42*; *p < 0.001*		*F = 31.54*; *p < 0.001*	
Parents’ Education Secondary Higher	143 (63.8%) 81 (36.2%)	89 (55.3%) 72 (44.7%)	76 (63.9%) 43 (36.1%)	68 (57.1%) 51 (42.9%)
	*Chi = 2.866 NS*		*Chi = 0.289 NS*	
Parents’ Job Employed Unemployed	37 (16.5%) 187 (83.5%)	148 (91.9%) 13 (8.1%)	22 (18.5%) 97 (81.5%)	108 (90.8%) 11 (9.2%)
	*Chi = 213.39*; *p < 0.001*		*Chi = 125.37*; *p < 0.001*	
Health Satisfied Less Satisfied	115 (51.3%) 109 (48.7%)	84 (52.2%) 77 (47.8%)	53 (44.5%) 66 (55.5%)	71 (59.7%) 48 (40.3%)
	*Chi Sq = 0.026 NS*		*Chi Sq 5.45*; *p < 0.05*	
Negative Moods Often Rarely	80 (35.7%) 144 (64.3%)	51 (31.7%) 110 (68.3%)	53 (44.5%) 66 (55.5%)	45 (37.8%) 74 (62.2%)
	*Chi Sq = 0.680 NS*		*Chi Sq = 1.11 NS*	
Marriage Satisfied Not Satisfied	131 (58.7%) 92 (41.3%)	124 (77.0%) 37 (23.0%)	69 (58.0%) 50 (42.0%)	81 (68.1%) 38 (31.9%)
	*Chi Sq 14.00*; *p < 0.001*		*Chi Sq 2.60 NS*	
Family Income Low Middle/High	70 (31.3%) 154 (68.8%)	47 (29.2%) 114 (70.8%)	30 (25.2%) 89 (74.8%)	
	*Chi Sq = 0.187 NS*			
Marriage Between Family Nonfamily	71 (31.7%) 153 (68.3%)	44 (27.3%) 117 (72.7%)	47 (39.5%) 72 (60.5%)	
	*Chi Sq = 0.853 NS*			
Children One Child Two Plus	84 (37.5%) 140 (62.5%)	50 (31.1%) 111 (68.9%)	54 (45.4%) 65 (54.6%)	
	*Chi Sq = 1.714 NS*			

**Table 2:** Child characteristics for unrelated and related parents with the statistical comparisons between mothers and fathers in the unrelated parent sample. NS = Not Significant.

Variable	Unrelated Parents		Related Parents (N = 119)
	Mothers (N = 224)	Fathers (N = 161)	
Child’s age in years:	Mean: 12.5 SD.: 5.01 Min: 4.00 Max: 44.00	Mean: 12.3 SD.: 3.47 Min.: 6.00 Max.: 23.00	Mean: 12.1 SD.: 4.34 Min.: 6 Max.: 34
	*F = 0.146 NS*		
Gender Male Female	170 (75.9%) 54 (24.1%)	131 (81.4%) 30 (18.6%)	90 (75.6%) 29 (24.4%)
	*Chi Sq = 1.645 NS*		
Communication Verbal Non-verbal	114 (50.9%) 110 (49.1%)	71 (44.1%) 90 (55.9%)	74 (62.2%) 45 (37.8%)
	*Chi Sq = 1.732 NS*		
Behaviour Problem Not problem	169 (75.4%) 55 (24.6%)	107 (66.5%) 54 (33.5%)	83 (69.7%) 36 (30.3%)
	*Chi Sq = 3.727 NS*		
Sensory Problem Not problem	103 (46.0%) 121 (54.0%)	57 (35.4%) 104 (64.6%)	52 (43.9%) 67 (56.1%)
	*Chi Sq = 4.316 NS*		
Severity Mild Moderate Severe	47 (21.0%) 112 (50.0%) 65 (29.0%)	35 (21.7%) 71 (44.1%) 55 (34.2%)	37 (31.1%) 55 (46.2%) 27 (22.7%)
	*Chi Sq = 1.506 NS*		

**Table 3:** Means and SDs of parental ratings on the measures used in the study and the results of statistical tests.

Measure	Unrelated Parents		Related Parents	
	Mothers (N = 224)	Fathers (N = 161)	Mothers (N = 119)	Fathers (N = 119)
Quality of Life	Mean: 70.9 SD.: 14.38	Mean: 74.0 SD.: 12.49	Mean: 71.7 SD.: 12.91	Mean: 76.1 SD.:14.14
	*F = 4.74*; *p < 0.05*		*F = 6.27*; *p < 0.05*	
Support Family	Mean: 37.0 SD.: 10.83	Mean: 38.1 SD.: 11.72	Mean: 37.4 SD.: 12.37	Mean: 36.7 SD.: 11.29
	*F = 0.895 NS*		*F = 0.21 NS*	
Support Friends	Mean: 15.1 SD.: 6.07	Mean: 14.2 SD.: 6.44	Mean: 15.1 SD.: 6.55	Mean: 14.6 SD.: 5.63
	*F = 1.96 NS*		*F = 0.433 NS*	
Professional Supports	Mean: 2.9 SD.: 1.28	Mean: 3.0 SD.: 1.34	Mean: 3.1 SD.: 1.28	Mean: 2.9 SD.: 1.28
	*F = 0.566 NS*		*F = 0.369 NS*	
Positive Stress	Mean: 16.9 SD.: 4.54	Mean: 17.2 SD.: 4.78	Mean: 16.7 SD.: 4.31	Mean: 16.8 SD.: 4.14
	*F = 0.408 NS*		*F = 0.034 NS*	
Negative Stress	Mean: 17.2 SD.: 4.55	Mean: 16.5 SD.: 4.92	Mean: 17.3 SD.: 4.30	Mean: 16.6 SD.: 4.66
	*F = 2.24 NS*		*F = 1.510 NS*	
Emotional Coping	Mean: 50.5 SD.: 10.29	Mean: 53.2 SD.: 11.10	Mean: 51.9 SD.: 11.12	Mean: 50.3 SD.: 11.39
	*F = 5.92*; *p < 0.05*		*F = 1.15 NS*	
Task Coping	Mean: 44.3 SD.: 7.43	Mean: 46.0 SD.: 8.22	Mean: 45.2 SD.: 7.18	Mean: 45.1 SD.: 7.15
	*F = 4.48*; *p < 0.05*		*F = 0.018 NS*	
Avoidance	Mean: 36.2 SD.: 8.50	Mean: 37.7 SD.: 10.13	Mean: 35.7 SD.: 9.57	Mean: 35.3 SD.: 7.37
	*F = 2.48 NS*		*F = 0.117 NS*	

NS = Not Significant; *p* > 0.05.

**Table 4:** (**a**) Correlations with quality-of-life measure and other measures. (**b**) The demographic characteristics of parents and children that were significantly associated with parental quality-of-life ratings for the two samples along with the statistical comparisons within unrelated and related parents. NS = Not Significant.

	Unrelated Parents (n = 385)		Related Parents (n = 238)	
**(a) Measure**	**Pearsons r**	**Significance**	**Pearsons r**	**Significance**
Support from Family	0.153	*p* < 0.01	0.235	*p* < 0.01
Support from Friends	0.124	*p* < 0.05	0.160	*p* < 0.05
Professional Supports	0.183	*p* < 0.010	0.130	*p* < 0.05
Positive Stress	0.180	*p* < 0.01	0.264	*p* < 0.01
Negative Stress	−0.132	*p* < 0.01	−0.228	*p* < 0.01
Emotional Coping	0.060	NS	0.059	NS
Task Coping	0.004	NS	−0.055	NS
Avoidance Coping	0.071	NS	0.197	*p* < 0.01
**(b) Predictor**	**Unrelated Parents (n = 385)**		**Related Parents (n = 238)**	
Parents’ Gender Mothers Fathers	Mean: 70.9 (SD 14.38) Mean: 74.0 (SD 12.49) *F = 4.74*; *p < 0.05*		Mean: 71.7 (SD 12.92) Mean: 76.1 (SD 14.14) *F = 6.27*; *p < 0.05*	
Parents’ Education Secondary Higher	Mean: 70.3 (SD 13.91) Mean: 75.0 (SD 12.90) *F = 11.01*; *p < 0.001*		Mean: 72.5 (SD 14.36) Mean: 76.1 (SD 12.36) *F = 3.977*; *p < 0.05*	
Parents’ job Employed Unemployed	Mean: 74.7 (SD 12.32) Mean: 69.9 (SD 14.51) *F = 11.924*; *p < 0.001*		Mean: 76.1 (SD 13.86) Mean: 71.2 (SD 13.06) *F = 7.642*; *p < 0.01*	
Health … Satisfied Less Satisfied	Mean: 76.8 (SD 12.86) Mean: 67.3 (SD 12.86) *F = 52.208*; *p < 0.001*		Mean: 79.7 (SD 12.29) Mean: 67.6 (SD 12.31) *F = 57.892*; *p < 0.001*	
Marriage … Satisfied Not Satisfied	Mean: 76.7 (SD 11.69) Mean: 63.3 (SD 13.12) F = 102.298; *p* < 0.001		Mean: 78.7 (SD 12.19) Mean: 65.7 (SD 12.17) F = 63.528; *p* < 0.001	
Child Behaviour Issues Yes No	Mean: 71.4 (SD 13.46) Mean: 74.1 (SD 14.14) *F = 3.045 NS*		Mean: 71.7 (SD 13.85) Mean: 78.9 (SD 11.69) *F = 14.393*; *p < 0.001*	
Child Sensory Issues Yes No	Mean: 70.2 (SD 14.13) Mean: 73.6 (SD 13.22) *F = 5.835*; *p < 0.05*		Mean: 70.9 (SD 13.79) Mean: 76.3 (SD 13.24) *F = 9.281*; *p < 0.005*	
Child Severity of Issues Mild Moderate Severe	Mean: 75.5 (SD 12.69) Mean: 72.7 (SD 13.43) Mean: 69.2 (SD 14.23) *F = 5.491*; *p < 0.005*		Mean: 76.3 (SD 14.85) Mean: 72.9 (SD 13.13) Mean: 72.3 (SD 12.86) *F = 1.811 NS*	

**Table 5:** (**a**) Linear regression on quality-of-life scores for related parents (N = 237). (**b**) Linear regression on quality-of-life scores for unrelated parents (N = 383). NS = Not Significant.

(a) Related Parents	Unstandardized Coefficients		Standardized Coefficients	t	Sig.
	B	Std. Error	Beta		
(Constant)	48.653	8.753		5.558	<0.001
Marriage Satisfaction	−9.210	1.516	−0.325	−6.074	<0.001
Satisfaction with Health	8.482	1.453	0.310	5.838	<0.001
Support from Family	0.224	0.081	0.193	2.773	0.006
Professional Supports	1.029	0.549	0.096	1.873	0.062
Positive Child-rearing	0.372	0.213	0.115	1.748	0.082
Educational Level	2.350	1.430	0.084	1.643	NS
Behavioural Problem	2.257	1.583	0.076	1.426	NS
Support from Friends	−0.085	0.155	−0.038	−0.552	NS
Negative	−0.096	0.199	−0.031	−0.483	NS
Avoidance Coping	0.113	0.088	0.070	1.281	NS
**(b) Unrelated Parents**	**Unstandardized Coefficients**		**Standardized Coefficients**	**t**	**Sig.**
	**B**	**Std. Error**	**Beta**		
(Constant)	59.361	6.412		9.258	<0.001
Marriage Satisfaction	−11.306	1.245	−0.390	−9.081	<0.001
Satisfaction with Health	8.301	1.167	0.304	7.112	<0.001
Educational Level	3.254	1.193	0.117	2.726	0.007
Professional Supports	1.175	0.446	0.112	2.636	0.009
Positive Child-rearing	0.312	0.137	0.106	2.283	0.023
Behavioural Problem	2.314	1.279	0.076	1.809	0.071
Support from Friends	0.197	0.113	0.090	1.744	0.082
Negative Stress	−0.122	0.138	−0.042	−0.886	NS
Support from Family	−0.048	0.066	−0.039	−0.723	NS
Avoidance Coping	0.000	0.066	0.000	−0.003	NS

## Data Availability

Anonymised datasets are available on reasonable request to the first author.
